# Surgical site infection after hip fracture – mortality and risk factors: an observational cohort study of 1,709 patients

**DOI:** 10.1080/17453674.2020.1717841

**Published:** 2020-01-24

**Authors:** Christian T Pollmann, Fredrik A Dahl, Jan Harald M Røtterud, Jan-Erik Gjertsen, Asbjørn Årøen

**Affiliations:** a Department of Orthopedic Surgery, Akershus University Hospital, Lørenskog;; b Institute of Clinical Medicine, Campus Ahus, University of Oslo, Oslo;; c Health Services Research Unit, Akershus University Hospital, Lørenskog;; d Norwegian Hip Fracture Register, Department of Orthopedic Surgery, Haukeland University Hospital, Bergen;; e Department of Clinical Medicine (K1), University of Bergen, Bergen;; f Department of Sports Medicine; Norwegian School of Sport Sciences, Oslo, Norway

## Abstract

Background and purpose — Surgical site infection (SSI) is a devastating complication of hip fracture surgery. We studied the contribution of early deep SSI to mortality after hip fracture surgery and the risk factors for deep SSI with emphasis on the duration of surgery.

Patients and methods — 1,709 patients (884 hemi­arthroplasties, 825 sliding hip screws), operated from 2012 to 2015 at a single center were included. Data were obtained from the Norwegian Hip Fracture Register, the electronic hospital records, the Norwegian Surveillance System for Antibiotic Use and Hospital-Acquired Infections, and the Central Population Register.

Results — The rate of early (≤ 30 days) deep SSI was 2.2% (38/1,709). Additionally, for hemiarthroplasties 7 delayed (> 30 days, ≤ 1 year) deep SSIs were reported. In patients with early deep SSI 90-day mortality tripled (42% vs. 14%, p < 0.001) and 1-year mortality doubled (55% vs. 24%, p < 0.001). In multivariable analysis, early deep SSI was an independent risk factor for mortality (RR 2.4 for 90-day mortality, 1.8 for 1-year mortality, p < 0.001). In univariable analysis, significant risk factors for early and delayed deep SSI were cognitive impairment, an intraoperative complication, and increasing duration of surgery. However, in the multivariable analysis, duration of surgery was no longer a significant risk factor.

Interpretation — Early deep SSI is an independent risk factor for 90-day and 1-year mortality after hip fracture surgery. After controlling for observed confounding, the association between duration of surgery and early and delayed deep SSI was not statistically significant.

Hip fractures, in usually frail, elderly patients, have high mortality rates of around 9% within 30 days (Sheikh et al. 2017) and up to 30% within 1 year (Lund et al. 2014). If a deep surgical site infection (SSI) ensues, a 1-year mortality rate of 50% (Edwards et al. 2008) has been reported. However, it is not clear to what extent this increased mortality rate is due to the infection and the treatment thereof and to what extent it is due to a more pronounced frailty which predisposed these patients to SSI (Belmont et al. 2014).

Considering the serious consequences of SSI for hip fracture patients it is important to optimize modifiable risk factors. However, reported risk factors differ, ranging from operative delay to the lead surgeon’s experience, duration of surgery, choice of implant, and patient factors such as obesity (Harrison et al. 2012, Cordero et al. 2016, de Jong et al. 2017, Zajonz et al. 2019).

Duration of surgery is a risk factor commonly focused upon. However, the question remains as to whether longer duration of surgery increases the risk of SSI by prolonging exposure to possible bacterial contamination (Stocks et al. 2010) or if prolonged duration of surgery represents a surrogate parameter for a difficult procedure or a complication as the main cause for an increased risk of SSI.

In this observational cohort study, we investigated the contribution of early deep SSI to mortality after hip fracture surgery and risk factors for early and delayed deep SSI in hip fracture patients with particular emphasis on the role of duration of surgery.

## Patients and methods

### Patients

**Figure 1.**
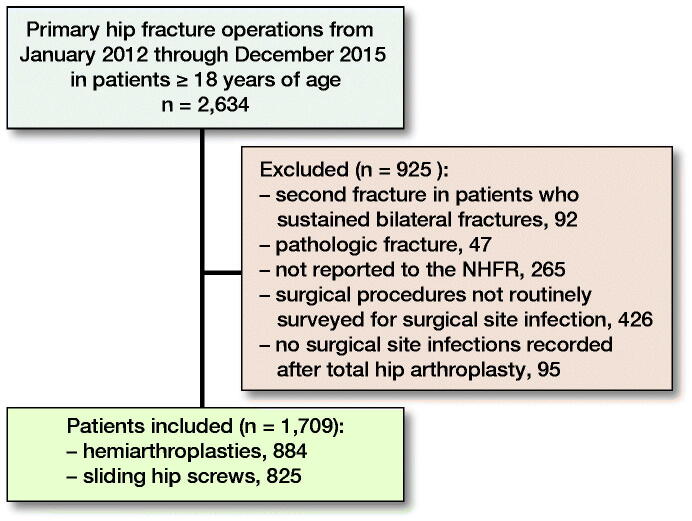
Flowchart of patient inclusion. NHFR: Norwegian Hip Fracture Register.

All patients 18 years of age or older who were operated with a hemiarthroplasty or a sliding hip screw for a non-pathologic fracture of the proximal femur at a single institution (Akershus university hospital [AUH]) from January 2012 through December 2015 and who were reported to the Norwegian Hip Fracture Register (NHFR) (Gjertsen et al. 2008) were included in this study (Figure 1). In patients who sustained 2 hip fractures during the study period (n = 92), only the 1st fracture was included in the analyses.

Other data from the present cohort of hip fracture patients have previously been used in an observational study on the effect of fast-track hip fracture care on mortality (Pollmann et al. 2019).

### Data collection

In Norway, hip fracture operations are prospectively reported to the NHFR (Gjertsen et al. 2008) by the surgeon on a 1-page questionnaire, which includes information on the time elapsed from fracture to surgery, cognitive impairment (“no”, “uncertain,” “yes”), ASA score, type of fracture, type of operation, type of anesthesia, pathological fractures, intraoperative complications (“no”/”yes” with supplemental free text), duration of surgery (time from incision to skin closure), and the surgeon’s experience (at least 1 surgeon present with > 3 years of experience in hip fracture surgery; “yes”/”no”). Using the unique 11-digit Norwegian personal identification number data from the NHFR and the electronic hospital records were linked deterministically.

### Surgical site infection

SSIs after hemiarthroplasty and total arthroplasty of the hip are surveyed under the Norwegian Surveillance System for Antibiotic Use and Hospital-Acquired Infections (NOIS) with 30-day and 1-year follow-up. A questionnaire is sent to each patient or, in the case of cognitive impairment or institutionalization, to the primary health care provider. If the patient reports an SSI or a suspicion of SSI this has to be confirmed by a physician on the same questionnaire. In equivocal cases the electronic hospital records are scrutinized and/or the primary health care provider is contacted. Until 2014, cases of SSI were defined according to the American Centers for Disease Control and Prevention (Horan et al. 2008) while from 2014 onwards case definitions from the European Centre for Disease Prevention and Control have been applied (Dalli 2012). Concerning SSIs, both definitions are practically identical. Sliding hip screws are not monitored by NOIS, but the Department of Microbiology and Infection Control at AUH also surveys SSIs with 30-day follow-up in these patients using the same method and criteria. The completeness of follow-up was 99%. In this study, SSI within 30 days of the index operation is termed early SSI, while an SSI diagnosed between 30 days and 1 year from the index operation is termed delayed SSI.

### Mortality

Mortality data from the Central Population Register are routinely imported into the electronic hospital records. There was no loss to follow-up regarding mortality. Mortality rates were calculated from the time of arrival at the hospital. Survival was censored at 1 year.

### Antibiotic prophylaxis

Fixation with antibiotic-loaded bone cement (0.5 g gentamicin per 40 g cement) was used in all hemiarthroplasties. All patients received perioperative systemic antibiotic prophylaxis.

### Statistics

Fisher’s exact test was used for unadjusted comparisons of proportions.

**Figure 2.**
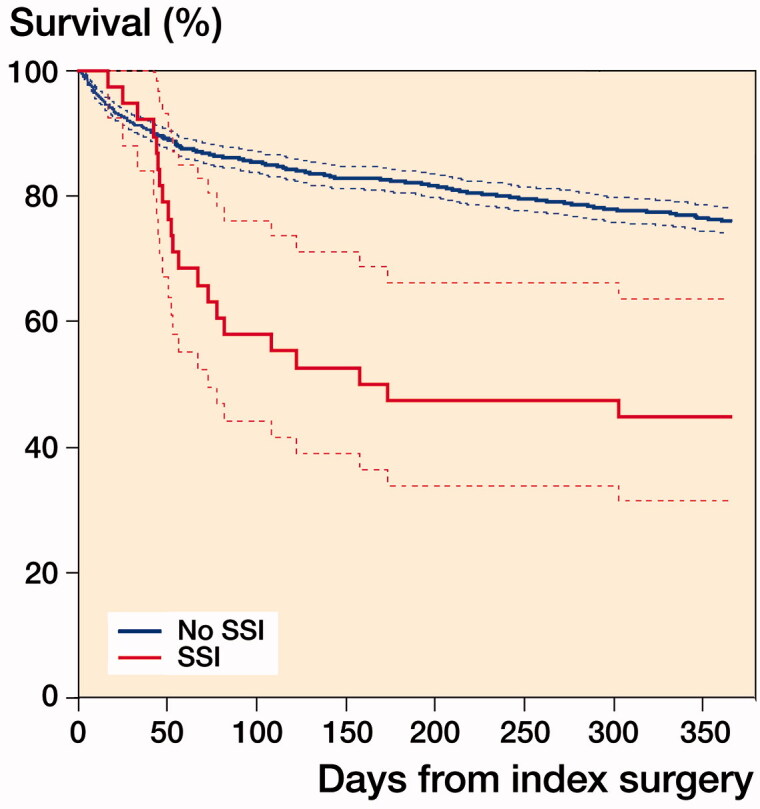
Kaplan–Meier patient survival curves with 95% confidence intervals for patients with and without early deep surgical site infection. SSI: early deep surgical site infection.

Due to the relatively high mortality rates we chose risk ratios as the statistical effect measure (Davies et al. 1998) for the multivariable model analyzing the effect of early deep SSI on mortality. Since log-binomial regression did not converge, Poisson regression with robust variance was chosen as the statistical model (Barros and Hirakata 2003). Risk ratios (RR) are presented with 95% confidence intervals (CI). We considered survival analysis by Cox regression. However, the Schoenfeld residuals ph-test showed that the proportional hazards assumption was not met, which is also illustrated by the Kaplan–Meier survival curve (Figure 2). Age squared was not a significant risk factor for mortality indicating that the effect of age on mortality was linear in our cohort. Age was therefore included as a continuous variable in the regression models. The type of intraoperative complication, specified as free text, ranged widely from myocardial infarction to technical problems to nausea and vomiting, to name a few. Therefore, we made no attempt at further classification and intraoperative complication was treated as a binary variable. Both the ASA score (≤ 2/≥ 3) and time from fracture to surgery (≤ 24 hours/> 24 hours) were dichotomized and included as binary variables.

Since there were relatively few cases of SSI, the analysis of risk factors for SSI was based on both early (sliding hip screws and hemiarthroplasties) and delayed (hemiarthroplasties only) SSIs to achieve a more robust statistical analysis. Logistic regression was used to analyze the risk factors for early and delayed deep SSI.

In all multivariable models, the variables to be adjusted for were chosen from directed acyclic graphs (DAG), which were constructed using DAGitty (Textor et al. 2017).

We performed a sensitivity analysis for the effect of early deep SSI on mortality by calculating the E-value. “The E-value is the minimum strength of association on the risk ratio scale that an unobserved confounder would need to have with both the exposure and the outcome, above and beyond the measured covariates, to fully explain away a specific exposure–outcome association” (VanderWeele and Ding 2017).

A p-value < 0.05 was considered as statistically significant. Data were analyzed with the SPSS statistical package version 25.0.0.1 (IBM Corp, Armonk, NY, USA).

### Ethics, registration, funding, and potential conflicts of interest

The Regional Ethics Committee South East deemed this study not to require approval (reference number 2015/409). Data were collected and handled in accordance with the requirements of the local data protection officer. The study was exempt from consent to participate. The Norwegian Data Inspectorate has approved the registration of data in the NHFR.

The study was funded by research grants from Sophies Minde AS and from the Norwegian Orthopedic Association in cooperation with Heraeus and by the Department of Orthopedic Surgery, Akershus university hospital.

The authors declare no conflicts of interest.

## Results

Patient characteristics are presented in Table 1; baseline data on the primary surgical treatment are given in Table 2 (see Supplementary data).

**Table 1.** Patient characteristics. Values are n (%) unless otherwise specified

Factor	Entire cohort (n = 1,709)	Survivors at 90 days (n = 1,459)	Deceased at 90 days (n = 250)	No SSI (n = 1,664)	SSI (n = 45)
Age, mean (SD)	82 (9.5)	81 (9.7)	85 (7.5)	82 (9.5)	81 (8.6)
Female sex	1,166 (68)	1,019 (70)	147 (59)	1,138 (68)	28 (62)
ASA					
1	27 (1.6)	27 (1.9)	–	27 (1.6)	–
2	451 (26)	429 (29)	22 (8.8)	444 (27)	7 (16)
3	1,021 (60)	865 (59)	156 (62)	988 (59)	33 (73)
4	171 (10)	104 (7.1)	67 (27)	166 (10)	5 (11)
5	3 (0.2)	1 (0.1)	2 (0.8)	3 (0.2)	–
Not reported	36 (2.1)	33 (2.3)	3 (1.2)	36 (2.2)	–
Cognitive impairment					
No	1113 (65)	1009 (69)	104 (42)	1091 (66)	22 (49)
Uncertain	178 (10)	143 (9.8)	35 (14)	169 (10)	9 (20)
Yes	374 (22)	267 (18)	107 (43)	362 (22)	12 (27)
Not reported	44 (2.6)	40 (2.7)	4 (1.6)	42 (2.5)	2 (4.4)
Type of fracture					
Femoral neck					
undisplaced	68 (4.0)	62 (4.2)	6 (2.4)	64 (3.8)	4 (8.9)
displaced	806 (47)	683 (47)	123 (49)	782 (47)	24 (53)
Basocervical	54 (3.2)	45 (3.1)	9 (3.6)	54 (3.2)	–
Trochanteric					
2 fragments	341 (20)	301 (21)	40 (16)	334 (20)	7 (16)
> 2 fragments	345 (20)	282 (19)	63 (25)	337 (20)	8 (18)
Intertrochanteric	39 (2.3)	34 (2.3)	5 (2.0)	38 (2.3)	1 (2.2)
Subtrochanteric	33 (1.9)	32 (2.2)	1 (0.4)	32 (1.9)	1 (2.2)
Other	19 (1.1)	16 (1.1)	3 (1.2)	19 (1.1)	–
Not reported	4 (0.2)	4 (0.3)	–	4 (0.2)	–

Percentages are column percentages;

SSI: early (sliding hip screws) and early and delayed (hemiarthroplasties) deep surgical site infection.

**Table 3.** Mortality (% and 95% CI) with and without early deep surgical site infection

Mortality	No SSI (n = 1,671)	Early deep SSI (n = 38)	Between-group difference	p-value^a^
30-day	8.3 (7.1–9.8)	5.3 (0.9–19)	–3.0 (–12 to 5.5)	0.8
90-day	14 (12–156)	42 (27–59)	28 (11 to 45)	< 0.001
1-year	24 (22–26)	55 (39–71)	31 (14 to 49)	< 0.001

SSI: surgical site infection.

a Fisher’s exact test.

### Surgical site infection

The rate of early SSI for all included procedures (hemiarthroplasties and sliding hip screws) during the study period was 2.2% (38/1,709) with a variation of between 0.5% and 3.1% per calendar year. For hemiarthroplasties the rate of early SSI was 2.4% (21/884) while it was 2.1% (17/825) for sliding hip screws. The cumulative 1-year SSI rate (early and delayed) for hemiarthroplasties was 3.2% (28/884). All SSIs were classified as deep and all but 1 patient with an infected hemiarthroplasty, who declined surgical treatment, were reoperated due to the SSI.

### Early deep surgical site infection and mortality

30-day mortality did not differ statistically significantly between patients with or without early deep SSI (Table 3). However, 90-day mortality tripled and 1-year mortality more than doubled in patients with early deep SSI (Table 3). A Kaplan–Meier cumulative survival curve illustrates that the increased mortality in patients with early deep SSI becomes apparent from approximately 6 weeks postoperatively (Figure 2).

The analysis of the causal association between early deep SSI and mortality was based on a DAG (Figure 3a, see Supplementary data) and confounders to be adjusted for were chosen from this DAG (Figure 3b, see Supplementary data). In this model, obesity, diabetes mellitus, and smoking represent unobserved confounders. In a multivariable analysis adjusted for age, sex, cognitive impairment, ASA score, the occurrence of an intraoperative complication, and time from fracture to surgery, early deep SSI was an independent risk factor for both 90-day and 1-year mortality (Table 4).

**Figure 4b.**
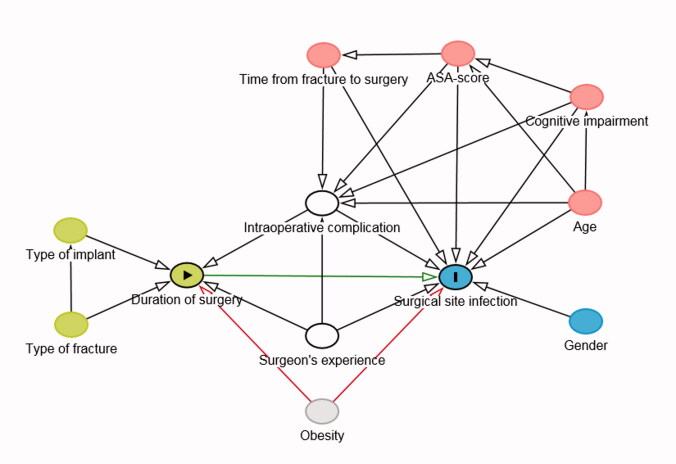
Directed acyclic graph depicting the adjustment for observed confounding of the association between duration of surgery and early and delayed deep surgical site infection.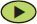 exposure; 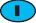 outcome; 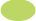 ancestor of exposure; 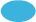 ancestor of outcome; 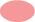 ancestor of exposure *and* outcome (confounder);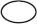 adjusted variable; 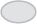 unobserved; 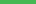 causal path; 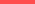
biasing path.

**Table 4.** Multivariable Poisson regression with robust variance of independent risk factors for 90-day and 1-year mortality

	90-day mortality		1-year mortality	
Factor	Risk ratio (CI)	p-value	Risk ratio (CI)	p-value
Age	1.04 (1.02–1.06)	< 0.001	1.03 (1.02–1.04)	< 0.001
Male sex	1.7 (1.4–2.2)	< 0.001	1.5 (1.3–1.8)	< 0.001
Cognitive impairment				
uncertain	1.5 (1.1–2.2)	0.02	1.3 (1.0–1.7)	0.05
yes	2.2 (1.7–2.8)	< 0.001	1.8 (1.5–2.2)	< 0.001
ASA score ≥ 3	3.0 (1.9–4.8)	< 0.001	2.5 (1.9–3.5)	< 0.001
Intraoperative				
complication	1.1 (0.7–1.6)	0.7	1.2 (1.0–1.6)	0.1
Time from fracture to				
surgery > 24 h	1.2 (0.9–1.5)	0.2	1.0 (0.8–1.2)	0.9
Early deep SSI	2.4 (1.6–3.5)	< 0.001	1.8 (1.3–2.5)	< 0.001

SSI: surgical site infection; 9.8% missing.

Omitting the variables intraoperative complication and time from fracture to surgery reduces missing cases from 9.8% to 3.9% while the parameter estimates for the remaining variables remain practically unchanged.

For the association between early deep SSI and mortality the E-values for the point estimate of the RR and for the lower bound of its CI were 4.2 and 2.6 for 90-day and 3.0 and 1.9 for 1-year mortality. Hence, an unobserved confounder that is associated with both early deep SSI and 90-day mortality by an RR of 4.2 each could explain away the observed RR of 2.4. An unobserved confounder that is associated with both early deep SSI and 90-day mortality by an RR of 2.6 each could move the lower bound of the CI to 1 (VanderWeele and Ding 2017).

### Risk factors for early and delayed deep surgical site infection

In a univariable analysis, cognitive impairment, the occurrence of an intraoperative complication, and longer duration of surgery were statistically significantly associated with an increased risk of early and delayed deep SSI (Table 5, see Supplementary data). An ASA score ≥ 3 bordered on being a statistically significant risk factor for early and delayed deep SSI (Table 5, see Supplementary data).

### Duration of surgery and early and delayed deep surgical site infection

The analysis of the causal association between duration of surgery and early and delayed deep SSI was based on a DAG (Figure 4a, see Supplementary data). Figure 4b shows which variables have to be adjusted for to control for observed confounding. Obesity represents an unobserved confounder.

In a multivariable analysis adjusted for the occurrence of an intraoperative complication and for surgeon’s experience the association between duration of surgery and early and delayed deep SSI is no longer statistically significant (Table 6, see Supplementary data).

## Discussion

### Early deep surgical site infection and mortality

In our cohort, 90-day mortality tripled and 1-year mortality more than doubled in patients with early deep SSI compared with patients without SSI.

In multivariable analysis, early deep SSI was an independent and important risk factor for both 90-day and 1-year mortality. Adjusting for age, cognitive impairment, and ASA score controls for a large part of frailty and the fact that SSI remained an independent risk factor for mortality indicates that SSI in itself increases the mortality rate.

The 30-day mortality rate did not differ statistically significantly between patients with early deep SSI and without SSI in our cohort and was in fact slightly lower in patients with early deep SSI. Edwards et al. (2008) observed a similar phenomenon. As the authors pointed out, this observation might have been caused by survival bias. The increase in 1-year mortality in our cohort was similar to the one reported by Merrer et al. (2007) (50% vs. 20%) and by Edwards et al. (2008) (50% vs. 30%).

The rate of deep SSI in our cohort was in the mid-range of earlier reported rates (Harrison et al. 2012, Sprowson et al. 2016, de Jong et al. 2017). Interestingly, we observed only deep SSIs as opposed to most other studies that report both deep and superficial SSIs (Merrer et al. 2007, Edwards et al. 2008, de Jong et al. 2017). What caused this discrepancy is unclear. Superficial SSIs may have been underreported in our cohort. Another possible explanation might be a difference in treatment strategy. It can be difficult to ascertain that an SSI is purely superficial and we might have a more aggressive approach revising SSIs that others might classify as superficial. Since a diagnosis of deep SSI made by the surgeon is one of the possible criteria that define a deep SSI (Horan et al. 2008, Dalli 2012), an aggressive revision policy could partly explain why no superficial SSIs were reported.

### Risk factors for early and delayed deep surgical site infection

In the univariable analysis cognitive impairment, the occurrence of an intraoperative complication and an increasing duration of surgery were statistically significantly associated with an increased risk of early and delayed deep SSI, while the association with an ASA score ≥ 3 bordered on statistical significance.

For clinical practice it is most relevant to identify modifiable risk factors for SSI. Pre-existing cognitive impairment can be considered non-modifiable, while delirium, which has a high incidence amongst hip fracture patients (Watne et al. 2014), and therefore probably accounts for some of the reported cognitive impairment in our cohort, may be preventable in some patients. A high ASA score might be modifiable if it is due to an acute condition or an acute deterioration of an existing ailment. However, most often the ASA score will not be modifiable. Some intraoperative complications may be preventable with adequate preoperative planning and experienced staff; however, it is in the nature of complications that not all of them can be prevented or even foreseen. An association between a longer duration of surgery and SSI has been shown before in several other studies (Harrison et al. 2012, Daley et al. 2015, Cheng et al. 2017, de Jong et al. 2017). On this basis, some authors have advocated measures to reduce duration of surgery (Cheng et al. 2017), such as expeditious surgical technique (Daley et al. 2015). However, the question remains how much of this association is due to the prolonged exposure to possible microbial contamination (Stocks et al. 2010) and how much is due to a longer duration of surgery being an indicator of a more complex surgical procedure, an inexperienced surgeon, or an intraoperative complication. To try to approach this question, we used a DAG with duration of surgery as the exposure and SSI as the outcome. From this DAG we determined that controlling for the occurrence of an intraoperative complication and for surgeon’s experience would control for all the observed confounders in our cohort. In the corresponding logistic regression model duration of surgery was no longer an independent risk factor for SSI. The fact that controlling for the occurrence of an intraoperative complication and for surgeon’s experience eliminated the statistical significance of the duration of surgery cannot readily be interpreted as duration of surgery not having a direct influence on the risk of SSI. However, this finding highlights the uncertainty that the prolonged exposure to possible bacterial contamination is the main reason for an association between duration of surgery and SSI. De Jong et al. (2017) reported an increased risk of SSI after hemiarthroplasty of the hip for both short (< 45 minutes) and long (> 90 minutes) durations of surgery. This might support the notion that careless tissue handling (short durations of surgery) and intraoperative complications (long durations of surgery) might play an important role in the development of SSI.

Our study has several strengths. With 1,709 patients the studied cohort is quite large. By using data from the NHFR, NOIS, the electronic hospital records, and the Central Population Register the cohort was well characterized. The loss to follow-up for SSI was small and no patients were lost to follow-up concerning mortality.

The study also has limitations. It is a single-center study. However, approximately 8% of all hip fracture operations in Norway are performed at our institution making this a relevant sample of Norwegian hip fracture patients. The number of cases of SSI was small (38 early SSIs, 7 delayed SSIs), limiting the number of covariates that could be included in and the statistical power of the multivariable regression model analyzing the risk factors for SSI. Information on delayed SSIs was only available for patients operated with a hemiarthroplasty.

Data on patients’ comorbidities was restricted to the ASA score and cognitive impairment, while no information was available on some known risk factors for SSI, such as diabetes mellitus (Tsang and Gaston 2013), obesity (Zajonz et al. 2019), or smoking (Durand et al. 2013). Obesity, in particular, represents an unobserved confounder in the association between duration of surgery and SSI. However, for the association between early deep SSI and mortality the E-values indicate that the evidence for causality is rather robust (VanderWeele and Ding 2017).

The variable “intraoperative complication” comprises a wide range of different events, which makes a detailed analysis impossible.

While a DAG can help to decide which variables to include in an analysis, it will always represent a simplification of reality.

In conclusion, our results indicate that an early deep SSI has a clinically significant impact on mortality in hip fracture patients and, hence, that the prevention of SSI should be seen as an essential aspect of hip fracture treatment. While we found no easily modifiable risk factors for early and delayed deep SSI in our cohort, we highly recommend adherence to the existing guidelines for the prevention of SSI (Ban et al. 2017). Additional measures, such as the use of high-dose dual-impregnated antibiotic-loaded bone cement in hemiarthroplasties (Sprowson et al. 2016) might be considered. We question the common wisdom that a longer duration of surgery in itself is closely associated with an increased risk of SSI and suggest that the underlying reason for a longer duration of surgery might be at least equally as important.

## Supplementary Material

Supplemental Material

## Supplementary data

Tables 2, 5, and 6 and Figures 3 and 4a are available as supplementary data in the online version of this article, http://dx.doi.org/10.1080/17453674.2020.1717841

## References

[CIT0001] Ban K A, Minei J P, Laronga C, Harbrecht B G, Jensen E H, Fry D E, Itani K M F, Dellinger E P, Ko C Y, Duane T M. Executive dummary of the American College of Surgeons/Surgical Infection Society surgical site infection guidelines—2016 update. Surg Infect 2017; 18(4): 379–82. 10.1089/sur.2016.214 28541808

[CIT0002] Barros A J, Hirakata V N. Alternatives for logistic regression in cross-sectional studies: an empirical comparison of models that directly estimate the prevalence ratio. BMC Med Res Methodol 2003; 3: 21. 14567763 10.1186/1471-2288-3-21 PMC521200

[CIT0003] Belmont P J, Garcia E S J, Romano D, Bader J O, Nelson K J, Schoenfeld A J. Risk factors for complications and in-hospital mortality following hip fractures: a study using the National Trauma Data Bank. Arch Orthop Trauma Surg 2014; 134(5): 597–604. 24570142 10.1007/s00402-014-1959-y

[CIT0004] Cheng H, Chen B P, Soleas I M, Ferko N C, Cameron C G, Hinoul P. Prolonged operative duration increases risk of surgical site infections: a systematic review. Surg Infect 2017; 18(6): 722–35. 10.1089/sur.2017.089 PMC5685201 28832271

[CIT0005] Cordero J, Maldonado A, Iborra S. Surgical delay as a risk factor for wound infection after a hip fracture. Injury 2016; 47(Suppl. 3): S56–S60. 27692108 10.1016/S0020-1383(16)30607-6

[CIT0006] Daley B J, Cecil W, Clarke P C, Cofer J B, Guillamondegui O D. How slow is too slow? Correlation of operative time to complications: an analysis from the Tennessee Surgical Quality Collaborative. J Am Coll Surg 2015; 220(4): 550–8. 25728140 10.1016/j.jamcollsurg.2014.12.040

[CIT0007] Dalli J. Case definitions of communicable diseases and special health issues. Brussels: European Commission; 2012. Available from: https://www.fhi.no/globalassets/dokumenterfiler/helseregistre/nois/ecdcs-kasusdefinisjoner-fullstendig-engelsk-versjon-av-kasusdefinisjonene-av-smittsomme-sykdommer-august-2012-pdf-.pdf (Accessed: 4 September 2019).

[CIT0008] Davies H T, Crombie I K, Tavakoli M. When can odds ratios mislead? BMJ 1998; 316(7136): 989–91. 9550961 10.1136/bmj.316.7136.989 PMC1112884

[CIT0009] de Jong L, Klem T, Kuijper T M, Roukema G R. Factors affecting the rate of surgical site infection in patients after hemiarthroplasty of the hip following a fracture of the neck of the femur. Bone Joint J 2017; 99-b(8): 1088–94. 28768787 10.1302/0301-620X.99B8.BJJ-2016-1119.R1

[CIT0010] Durand F, Berthelot P, Cazorla C, Farizon F, Lucht F. Smoking is a risk factor of organ/space surgical site infection in orthopaedic surgery with implant materials. Int Orthop 2013; 37(4): 723–7. 23443979 10.1007/s00264-013-1814-8 PMC3609989

[CIT0011] Edwards C, Counsell A, Boulton C, Moran C G. Early infection after hip fracture surgery: risk factors, costs and outcome. J Bone Joint Surg Br 2008; 90(6): 770–7. 18539671 10.1302/0301-620X.90B6.20194

[CIT0012] Gjertsen J E, Engesaeter L B, Furnes O, Havelin L I, Steindal K, Vinje T, Fevang J M. The Norwegian Hip Fracture Register: experiences after the first 2 years and 15,576 reported operations. Acta Orthop 2008; 79(5): 583–93. 18839363 10.1080/17453670810016588

[CIT0013] Harrison T, Robinson P, Cook A, Parker M J. Factors affecting the incidence of deep wound infection after hip fracture surgery. J Bone Joint Surg Br 2012; 94(2): 237–40. 22323693 10.1302/0301-620X.94B1.27683

[CIT0014] Horan T C, Andrus M, Dudeck M A. CDC/NHSN surveillance definition of health care-associated infection and criteria for specific types of infections in the acute care setting. Am J Infect Control 2008; 36(5): 309–32. 18538699 10.1016/j.ajic.2008.03.002

[CIT0015] Lund C A, Moller A M, Wetterslev J, Lundstrom L H. Organizational factors and long-term mortality after hip fracture surgery: a cohort study of 6143 consecutive patients undergoing hip fracture surgery. PLoS One 2014; 9(6): e99308. 24926876 10.1371/journal.pone.0099308 PMC4057264

[CIT0016] Merrer J, Girou E, Lortat-Jacob A, Montravers P, Lucet J C. Surgical site infection after surgery to repair femoral neck fracture: a French multicenter retrospective study. Infect Control Hosp Epidemiol 2007; 28(10): 1169–74. 17828694 10.1086/520745

[CIT0017] Pollmann C T, Rotterud J H, Gjertsen J E, Dahl F A, Lenvik O, Aroen A. Fast track hip fracture care and mortality: an observational study of 2230 patients. BMC Musculoskelet Disord 2019; 20(1): 248. 31122228 10.1186/s12891-019-2637-6 PMC6533651

[CIT0018] Sheikh H Q, Hossain FS, Aqil A, Akinbamijo B, Mushtaq V, Kapoor H. A comprehensive analysis of the causes and predictors of 30-day mortality following hip fracture surgery. Clin Orthop Surg 2017; 9(1): 10–8. 28261422 10.4055/cios.2017.9.1.10 PMC5334018

[CIT0019] Sprowson A P, Jensen C, Chambers S, Parsons N R, Aradhyula N M, Carluke I, Inman D, Reed M R. The use of high-dose dual-impregnated antibiotic-laden cement with hemiarthroplasty for the treatment of a fracture of the hip: the Fractured Hip Infection trial. Bone Joint J 2016; 98-b(11): 1534–41. 27803231 10.1302/0301-620X.98B11.34693 PMC5102031

[CIT0020] Stocks G W, Self S D, Thompson B, Adame X A, O’Connor D P. Predicting bacterial populations based on airborne particulates: a study performed in nonlaminar flow operating rooms during joint arthroplasty surgery. Am J Infect Control 2010; 38(3): 199–204. 19913327 10.1016/j.ajic.2009.07.006

[CIT0021] Textor J, van der Zander B, Gilthorpe M S, Liśkiewicz M, Ellison G T. Robust causal inference using directed acyclic graphs: the R package ‘Dagitty’. Int J Epidemiol 2017; 45(6): 1887–94. 10.1093/ije/dyw341 28089956

[CIT0022] Tsang S T, Gaston P. Adverse peri-operative outcomes following elective total hip replacement in diabetes mellitus: a systematic review and meta-analysis of cohort studies. Bone Joint J 2013; 95-b(11): 1474–9. 24151265 10.1302/0301-620X.95B11.31716

[CIT0023] VanderWeele T J, Ding P. Sensitivity analysis in observational research: introducing the E-value. Ann Intern Med 2017; 167(4): 268–74. 28693043 10.7326/M16-2607

[CIT0024] Watne L O, Torbergsen A C, Conroy S, Engedal K, Frihagen F, Hjorthaug G A, Juliebo V, Raeder J, Saltvedt I, Skovlund E, Wyller T B. The effect of a pre- and postoperative orthogeriatric service on cognitive function in patients with hip fracture: randomized controlled trial (Oslo Orthogeriatric Trial). BMC Med 2014; 12:63. 24735588 10.1186/1741-7015-12-63 PMC4022270

[CIT0025] Zajonz D, Brand A, Lycke C, Ozkurtul O, Theopold J, Spiegl U J A, Roth A, Josten C, Fakler J K M. Risk factors for early infection following hemiarthroplasty in elderly patients with a femoral neck fracture. Eur J Trauma Emerg Surg 2019; 45(2): 207–1. 29340736 10.1007/s00068-018-0909-8

