# Supplementary material for: Surgical site infection after hip fracture – mortality and risk factors: an observational cohort study of 1,709 patients
Source: Acta Orthop. 2020 Jan 24;91(3):347–52. doi: 10.1080/17453674.2020.1717841 (PMC8023915; doi:10.1080/17453674.2020.1717841)
Supplement: Supplemental Material [file IORT_A_1717841_SM7507.pdf]

Supplementary data

Table 2. Surgical treatment. Values are n (%) unless otherwise specified

Factor	Entire cohort (n = 1,709)	Survivors at 90 days (n = 1,459)	Deceased at 90 days (n = 250)	No SSI (n = 1,664)	SSI (n = 45)
Hours from fracture to surgery					
0–6	11 (0.6)	9 (0.6)	2 (0.8)	11 (0.7)	–
>6–12	115 (6.7)	105 (7.2)	10 (4.0)	113 (6.8)	2 (4.4)
>12–24	514 (30)	449 (31)	65 (26)	504 (30)	10 (22)
>24–48	698 (41)	590 (40)	108 (43)	678 (41)	20 (44)
>48	302 (18)	248 (17)	54 (22)	290 (17)	12 (27)
Not reported	69 (4.0)	58 (4.0)	11 (4.4)	68 (4.1)	1 (2.2)
Type of anesthesia					
Spinal	1,476 (86)	1,258 (86)	218 (87)	1,435 (86)	41 (91)
General	152 (8.9)	131 (9.0)	21 (8.4)	149 (9.0)	3 (6.7)
Other	59 (3.5)	51 (3.5)	8 (3.2)	58 (3.5)	1 (2.2)
Not reported	22 (1.3)	19 (1.3)	3 (1.2)	22 (1.3)	–
Type of procedure					
Hemiarthroplasty	884 (52)	753 (52)	131 (52)	856 (51)	28 (62)
Sliding hip screw	825 (48)	706 (48)	119 (48)	808 (49)	17 (38)
Surgeon's experience					
≤ 3 years	209 (12)	176 (12)	33 (13)	203 (12)	6 (13)
> 3 years	1427 (84)	1219 (84)	208 (83)	1,388 (83)	39 (87)
Not reported	73 (4.3)	64 (4.4)	9 (3.6)	73 (4.4)	–
Intraoperative complication					
No	1,524 (89)	1,305 (89)	219 (88)	1,487 (89)	37 (82)
Yes	126 (7.4)	103 (7.1)	23 (9.2)	119 (7.2)	7 (16)
Not reported	59 (3.5)	51 (3.5)	8 (3.2)	58 (3.5)	1 (2.2)
Duration of surgery [a]					
median	64	65	60	64	65
range	16–241	16–241	19–144	16–209	30–241

Percentages are column percentages; SSI: early (sliding hip screws) and early and delayed (hemiarthroplasties) deep surgical site infection;
[a] Time from incision to skin closure in minutes.

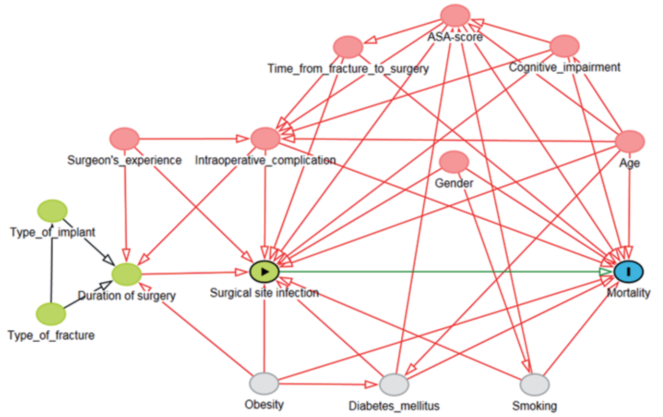

Figure 3a. Directed acyclic graph depicting a causal model for the association between early deep surgical site infection and mortality. exposure; outcome; ancestor of exposure; ancestor of exposure *and* outcome (confounder); unobserved; causal path; biasing path.

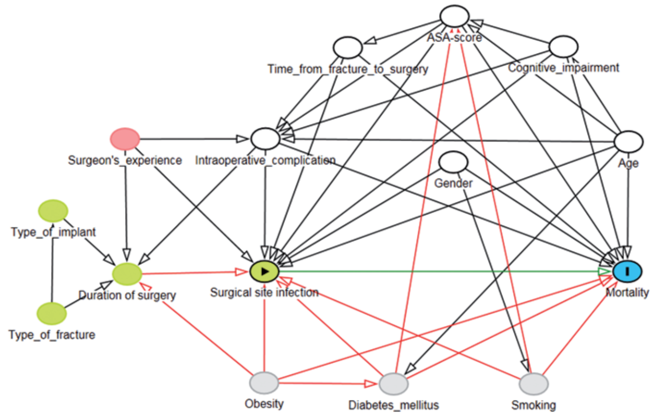

Figure 3b. Directed acyclic graph depicting the adjustment for observed confounding of the association between early deep surgical site infection and mortality. For symbols, see Figure 3a and adjusted variable.

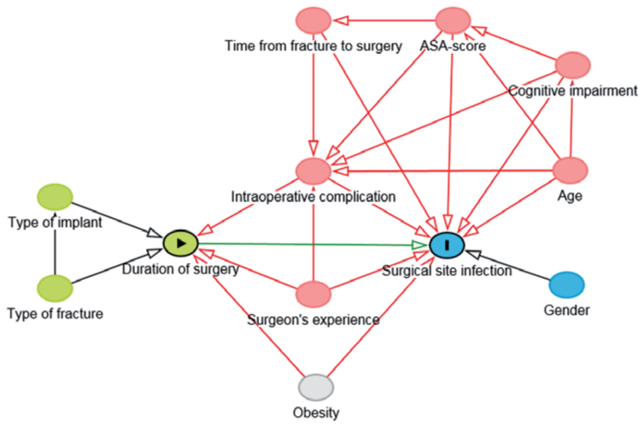

Figure 4a. Directed acyclic graph depicting a causal model for the association between duration of surgery and early and delayed deep surgical site infection. (green ellipse) exposure; (blue ellipse) outcome; (green) ancestor of exposure; (blue) ancestor of outcome; (red) ancestor of exposure *and* outcome (confounder); (grey) unobserved; (green line) causal path; (red line) biasing path.

Table 5. Univariable binary logistic regression analysis of risk factors for early and delayed deep surgical site infection

Factor	Early and delayed deep SSI [a] Odds ratio (CI)	p-value	Missing (%)
Cognitive impairment		0.05	2.6
uncertain	2.6 (1.2–5.8)	0.02	
yes	1.6 (0.8–3.4)	0.2	
Intraoperative complication	2.4 (1.03–5.4)	0.04	3.5
Duration of surgery (hours)	1.9 (1.1–3.5)	0.04	–
ASA score ≥ 3	2.2 (0.98–5.0)	0.06	2.1

[a] Early (sliding hip screws) and early and delayed (hemiarthroplasties) deep surgical site infection.
ASA: American Society of Anesthesiologists.

Table 6. Multivariable binary logistic regression analysis of the association between duration of surgery and early and delayed deep surgical site infection

Factor	Early and delayed deep SSI [a] Odds ratio (CI)	p-value
Duration of surgery (hours)	1.5 (0.8–2.9)	0.2
Intraoperative complication	2.1 (0.9–4.9)	0.1
Surgeon's experience [b]	0.9 (0.4–2.2)	0.9

[a] Early (sliding hip screws) and early and delayed (hemiarthroplasties) deep surgical site infection.
[b] At least 1 surgeon with more than 3 years of experience in hip fracture surgery; 7.1% missing.
